# Supplementary material for: Identifying chemicals associated with irritable bowel syndrome by integrating a transcriptome-wide association study with chemical-gene-interaction analysis
Source: PLoS One. 2026 Feb 3;21(2):e0340666. doi: 10.1371/journal.pone.0340666 (PMC12867268; doi:10.1371/journal.pone.0340666)
Supplement: Attachment — Submitted filename: Response to Reviewers.docx [file pone.0340666.s003.docx]

**Zhao Xl et al. Manuscript PONE-D-25-32341R1**

**Identifying Chemicals Associated with Irritable Bowel Syndrome by Integrating a Transcriptome-Wide Association Study with Chemical-Gene-Interaction Analysis**

Reply to Editor and Reviewers:

Thank you very much for giving me the opportunity to revise the manuscript. We appreciate the reviewer’s strict scientific attitude and constructive suggestions. Thank you some much for all your contributaion on my research work. We have included a point by point response in this letter. Besides, we have submitted a revised manuscript.

Reviewer #1: 1.The abstract (28518 IBS cases) and section 2.1 (455321 total sample) have a logical break in data, and the screening process needs to be clearly explained.

2. The statistical correction method for chemical enrichment analysis (such as whether FDR is used) and the screening threshold basis (incomplete citation of standards with | NES |>1) are not specified.

3.Section 2.5 uses a confidence threshold of 0.15 for STRING database (much lower than the conventional 0.4), without explaining the rationale or conducting sensitivity validation.

4.Section 2.4 does not specify the control group design for differential gene screening (such as the experimental details of EGR3 overexpression group vs control group).

5. The Manhattan plot in Figure 1 does not indicate the corresponding organizational type (A-E) of the subgraph, and readers cannot associate the result description.

Figure 2C Venn diagram does not indicate the names of intersecting genes (only 5 genes are mentioned but not listed in the caption/text).

6.Section 3.5 selects 269 chemicals and only lists the top 50, lacking a classification summary (such as the distribution ratio of drugs/pollutants/heavy metals).

7. Verification queue limitations not declared

Response to Independent Review Report, Reviewer 1

**Question1:**: The abstract (28518 IBS cases) and section 2.1 (455321 total sample) have a logical break in data, and the screening process needs to be clearly explained.

**Response:** Thank you for these precious comments. We have revised the description of the cohort data to make the sample source more explicit. The detailed description is as follows:

2.1 GWAS Dat

The summary data for the IBS GWAS were acquired from previously published research [20]. In detail, the study utilized genotype and phenotype data from the European ancestry subset of the UK Biobank (UKB) cohort, which is a population-based volunteer longitudinal cohort of ~500,000 individuals recruited across 22 centers in the United Kingdom. This European ancestry subset included 456,327 individuals, among whom 348,441 were unrelated.

The IBS phenotype was defined based on multi-source data including death registers, self-reported records, hospital admission data, and primary care records (UKB data field: 131639). Initially, 29,524 participants were identified as original IBS cases; after excluding 1,006 participants who also had inflammatory bowel disease (IBD) diagnoses, 28,518 individuals were finally assigned to the IBS case group, with the remaining 426,803 participants in the European ancestry subset serving as controls.

Genotype data of the UKB cohort were imputed using the Haplotype Reference Consortium (HRC) and UK10K as reference samples. Genotype probabilities were transformed into definitive genotypes utilizing PLINK2 (hard-call 0.1). Single nucleotide polymorphisms (SNPs) were excluded from the analysis if they met any of the following criteria: minor allele count < 5, Hardy-Weinberg equilibrium test P-value < 1.0×10⁻⁵, missing genotype rate > 0.05, or imputation accuracy (Info) score < 0.3. Comprehensive details regarding subjects, genotyping procedures, imputation, and quality control measures can be referenced in the original study.

**Question2:** The statistical correction method for chemical enrichment analysis (such as whether FDR is used) and the screening threshold basis (incomplete citation of standards with | NES |>1) are not specified.

**Response:** As the reviewers' suggestions, we reapplied the FDR method to adjust the CGSEA results. We have updated the content in the Methods and Results sections as follows:

2.5 Protein-protein interaction (PPI)

PPI networks for TWAS-identified genes were constructed and visualized using Cytoscape software (version 3.9.0 or later), with PPI data retrieved from the STRING database (confidence score ≥ 0.4 as the cutoff) and visualization parameters (node size, edge thickness, color gradients) adjusted to distinguish gene/protein nodes and their interaction strengths[27]. Subsequently, the MCODE plugin was applied to the PPI network with optimized core parameters (degree cutoff = 2, node score cutoff = 0.2, k-core = 2, maximum depth from seed = 100) to cluster densely connected sub-networks, and the top 5 modules with the highest MCODE scores were selected as key functional clusters. Meanwhile, the CytoHubba plugin was used to screen hub genes by employing three complementary algorithms: Maximal Clique Centrality (MCC), which accurately identifies functionally critical core hub genes by considering both direct and indirect interactions via clique structures; Edge Percolated Component (EPC), which highlights nodes pivotal to pathway stability by evaluating contributions to network connectivity and robustness; and Degree, a computationally efficient and intuitive method for preliminary screening of hub genes with extensive direct interactions.

2.6 Chemical-related Gene Set Enrichment Analysis (CGSEA)

In this investigation, the chemical gene set enrichment analysis was conducted utilizing the CGSEA tool, which is based on previously published research [28]. The CGSEA tool serves to evaluate the correlations between chemicals and intricate diseases. We employed the CTD chemical-gene interaction network alongside TWAS expression associations to analyze the statistics related to Irritable Bowel Syndrome (IBS) in order to examine the connections between chemicals and diseases from a genomic functional standpoint. Additionally, we applied weighted Kolmogorov-Smirnov run sum statistics to further investigate the association between chemicals and IBS. The dataset for chemical-associated gene expression annotations utilized in this study was acquired from the Comparative Toxicogenomics Database (CTD) (http://ctdbase.org/downloads/), which includes 1,788,149 chemical-gene pair annotation terms, resulting in a compilation of 11,190 chemical-associated genes[29]. To address the issue of multiple testing bias inherent in high-throughput chemical-gene association analysis, we applied false discovery rate (FDR) correction to the CGSEA results in addition to the P-value filtering. The screening thresholds for significant chemicals were defined as follows: 1) P_CGSEA < 0.05 (primary association signal); 2) FDR < 0.05 (corrected for multiple comparisons); 3) |normalized enrichment score (NES)| > 1[30].

3.5 CGSEA of the TWAS-identified genes

We conducted a CGSEA analysis to explore the environmental factors affecting IBS, identifying 1,789 chemicals (Supplementary Information 2), including 112 significant chemicals (FDR< 0.05, |normalized enrichment score, NES|>1). Table 3 lists the top50 compounds ranked by NES. We classified 112 significant chemicals and mapped them to the genes identified by TWAS (Figure 5A). The classifications include Drugs, Nutrients, Organic Compounds, Pollutants/Heavy Metals/Carcinogens, Inorganic Compounds, and Pesticides. The number and proportion of compounds corresponding to each category are shown in Figure 5B. We further performed PPI (Protein-Protein Interaction) analysis on the TWAS-identified genes and used the MCODE plugin to identify 5 key MCODEs in the PPI network (Figure 5C). Additionally, we analyzed the hub genes in the network using the CytoHubba plugin (Figure 5D). Notably, HLA-DQA1 and HLA-DQA2 are not only hub genes but were also identified in our previous analysis.

**Question3:** Section 2.5 uses a confidence threshold of 0.15 for STRING database (much lower than the conventional 0.4), without explaining the rationale or conducting sensitivity validation.

**Response:** We sincerely appreciate the reviewers for pointing out our oversight. We have revised the confidence threshold of the STRING database from 0.15 to 0.4 and re-performed the PPI analysis using STRING. The resulting outcomes are consistent with the previous ones, which also indicates that our results are robust and have passed the sensitivity test. We have updated the content in the Methods and Results sections as follows:

2.5 Protein-protein interaction (PPI)

PPI networks for TWAS-identified genes were constructed and visualized using Cytoscape software (version 3.9.0 or later), with PPI data retrieved from the STRING database (confidence score ≥ 0.4 as the cutoff) and visualization parameters (node size, edge thickness, color gradients) adjusted to distinguish gene/protein nodes and their interaction strengths[27]. Subsequently, the MCODE plugin was applied to the PPI network with optimized core parameters (degree cutoff = 2, node score cutoff = 0.2, k-core = 2, maximum depth from seed = 100) to cluster densely connected sub-networks, and the top 5 modules with the highest MCODE scores were selected as key functional clusters. Meanwhile, the CytoHubba plugin was used to screen hub genes by employing three complementary algorithms: Maximal Clique Centrality (MCC), which accurately identifies functionally critical core hub genes by considering both direct and indirect interactions via clique structures; Edge Percolated Component (EPC), which highlights nodes pivotal to pathway stability by evaluating contributions to network connectivity and robustness; and Degree, a computationally efficient and intuitive method for preliminary screening of hub genes with extensive direct interactions.

**Question4:** Section 2.4 does not specify the control group design for differential gene screening (such as the experimental details of EGR3 overexpression group vs control group).

**Response:** We sincerely appreciate the reviewers for pointing out the inaccuracies in our description. We have revised the description in the Methods section as follows:

2.4 Gene functionalanalyses

GEO2R [23] was used to find DEGs between diarrhea-irritable bowel syndrome (d-IBS) patients and control groups. P value < 0.05 and |Log2FC| >1 were considered as differentially expressed genes (DEGs). Quality control was performed using principal component analysis (PCA) via the prcomp function and ggplot2 to verify sample grouping. The volcano plot and heatmap analysis were done using R packages[24][25].Functional enrichment analysis of DEGs was conducted with the clusterProfiler package for Gene Ontology (GO) and Kyoto Encyclopedia of Genes and Genomes (KEGG) pathways, with significant terms filtered by adjusted P-value < 0.05(Benjamini-Hochberg method for false discovery rate correction)[26].

**Question5:** The Manhattan plot in Figure 1 does not indicate the corresponding organizational type (A-E) of the subgraph, and readers cannot associate the result description.

Figure 2C Venn diagram does not indicate the names of intersecting genes (only 5 genes are mentioned but not listed in the caption/text).

**Response:** We sincerely appreciate the reviewers' suggestions. We have indicated the names of 5 intersecting genes in Figure 2. We have updated Figures 1 to enhance their visual appeal and revised the legends to improve readability, with the updated legend content as follows:

Figure 1. Manhattan plots of the association results from the whole transcriptome-wide association study. The horizontal line indicates PTWAS, with green dots indicating PTWASp<0.05 and red dots indicating PTWAS<0.01. Each point represents the genetic predicted expression of a specific gene by the prediction models for small intestine, colon transverse, colon sigmoid, whole blood, and peripheral blood tissues. The x-axis represents the genomic position of the corresponding gene, and the y-axis represents the negative logarithm of the association P-value.

**Question6:** Section 3.5 selects 269 chemicals and only lists the top 50, lacking a classification summary (such as the distribution ratio of drugs/pollutants/heavy metals).

**Response:** As the reviewers' suggestions, we have updated the classification of chemicals in Table 3.

**Question7:** Verification queue limitations not declared.

**Response:** We sincerely appreciate the reviewers for pointing out the inadequacy in our description of the study's limitations. We have revised the description regarding the Verification queue limitations as follows:
Our study explored a large number of compounds associated with IBS by analyzing gene-environment interactions, providing novel insights for IBS research. However, this study still has certain limitations. Firstly, the GWAS summary data used in this research is mainly derived from European populations, which means the findings may exhibit varying degrees of differences across different populations. The lack of multi-ethnic validation cohorts (e.g., Asian, African, or American populations) as well as independent clinical cohorts with detailed phenotypic records may restrict the generalizability of our genetic association results. Secondly, the transcriptomic data used to validate the identified IBS-related key genes was primarily obtained from newly diagnosed IBS-D patients who met the Rome III criteria, prospectively recruited at a gastroenterology clinic by Eloísa Salvo-Romero et al. [20]. The diagnosis of IBS is relatively challenging, resulting in a small number of patients, which may introduce bias into the validation results. Finally, the IBS-related key genes and compounds identified in this study require further validation in subsequent research, including functional experiments to elucidate their biological functions in the pathogenesis of IBS and their potential pathogenic or therapeutic value.

Reviewer #2: The study investigates the multifactorial etiology of irritable bowel syndrome (IBS) by integrating genetic and environmental analyses. Using GWAS data from more than 455,000 individuals, including over 28,000 IBS cases, the authors performed a transcriptome-wide association study (TWAS) supported by gene expression data from gastrointestinal and blood tissues. Functional enrichment and protein–protein interaction analyses highlighted relevant biological pathways, while chemical-related gene set enrichment analysis (CGSEA) identified 269 environmental chemicals associated with IBS. In total, 33 significant genes were detected, underscoring potential gene–environment interactions. Overall, the manuscript provides a valuable contribution by combining genetic and environmental perspectives to improve our understanding of IBS.

The background and motivation are well structured, and the availability of a large, previously published GWAS dataset strengthens the study. The methods used for TWAS, enrichment, and protein–protein interaction analysis are appropriate and consistent with similar studies. The discussion acknowledges demographic limitations and contextualizes the findings with relevant literature.

Major Concerns:

-The TWAS analysis requires further clarification. The rationale for using the FUSION model and GWAS summary data to infer gene expression should be explained, and the justification for including the additional GEO dataset is not fully clear.

-The statement regarding “33 significant unduplicated genes” alongside the listing of hundreds of genes per tissue is confusing. It is unclear what “unduplicated” means in this context (e.g., non-redundant across tissues, or differentially expressed?). This needs clearer explanation.

-If all genes are significant after FDR correction, the purpose of Figure 2.A is not obvious. The figure legends also require clarification—should the corrected genes across all five tissues amount to five overlapping genes, or ~1,120 combined? This discrepancy should be addressed.

-While enrichment analysis was performed on the common genes, a comparison of GO/KEGG enrichment for significant genes in each tissue individually would provide additional insights.

-Section 3.3 should specify the selection criteria for “enriched terms” and the types of “interactions” used in STRING. Figure 3, especially 3.A, is crowded and difficult to interpret; presenting results for common differentially expressed genes may improve clarity.

-In Section 3.4, TWAS and DEGs are reported to share 18 genes. It would be useful to contextualize this relative to the 1,120 TWAS genes, and to indicate their tissue distribution. Similarly, whether the five common TWAS genes are represented in the DEG results and how they are expressed should be clarified.

-The reported numbers of unique genes (1101 TWAS vs. 315 DEG) should be corrected and explained. The large differences may reflect tissue- or phenotype-specific factors, which merit discussion.

-Figures 4 and 5 require improvement. Figure 4.B shows a healthy sample clustering close to IBS samples—could this be due to normalization or batch effects? Was this also observed in the clustering analysis (Figure 4.D)? Figure 5 would be more informative if it included network analysis outputs such as hub genes or subgraphs.

-Table 3 could be enhanced by categorizing chemicals (e.g., drugs, nutrients), which would add interpretability.

-In the discussion, while drug families such as antipsychotics and anti-inflammatories are highlighted, the lack of enrichment for other drugs in the same families is not addressed. A mechanistic discussion comparing enriched vs. non-enriched drugs would strengthen the interpretation.

-The conclusion is relatively weak and would benefit from more detail, emphasizing the significance of the findings and potential directions for future work.

-Supplementary Information 1–2 was not available for review and should be provided.

Minor Concerns:

-Several typographical and formatting errors should be corrected. Examples include:

-Section title “2.4. Gene functionalanalyses” → “2.4. Gene functional analyses”

-Section 3.2 “IBSamong” → “3.2. IBS among”

-Section 3.4 “GSE14841DEGs” → “GSE14841 DEGs”

-Figure 4 legend “C: vocanol” → “C: volcano”

-A thorough proofreading will improve readability.

**Reviewer 2:** The study investigates the multifactorial etiology of irritable bowel syndrome (IBS) by integrating genetic and environmental analyses. Using GWAS data from more than 455,000 individuals, including over 28,000 IBS cases, the authors performed a transcriptome-wide association study (TWAS) supported by gene expression data from gastrointestinal and blood tissues. Functional enrichment and protein–protein interaction analyses highlighted relevant biological pathways, while chemical-related gene set enrichment analysis (CGSEA) identified 269 environmental chemicals associated with IBS. In total, 33 significant genes were detected, underscoring potential gene–environment interactions. Overall, the manuscript provides a valuable contribution by combining genetic and environmental perspectives to improve our understanding of IBS.

The background and motivation are well structured, and the availability of a large, previously published GWAS dataset strengthens the study. The methods used for TWAS, enrichment, and protein–protein interaction analysis are appropriate and consistent with similar studies. The discussion acknowledges demographic limitations and contextualizes the findings with relevant literature.

**Response:** Thank you for these precious comments concerning my manuscript. These comments are all valuable and very helpful for revising and improving my paper, as well as the important guiding significance to my researches. We have studied comments carefully and have made corrections which we hope meet with approval. The responds to the reviewer’s comments are as follows:

**Question1:** The TWAS analysis requires further clarification. The rationale for using the FUSION model and GWAS summary data to infer gene expression should be explained, and the justification for including the additional GEO dataset is not fully clear.

**Response:** We sincerely appreciate the reviewers for pointing out the inadequacies in the descriptions of our manuscript. We have revised the rationale for using the FUSION model and GEO dataset for validation as follows:

2.2 TWAS analysis

TWAS is a powerful method that integrates gene expression profiles with GWAS summary data. Compared with linkage disequilibrium-based (LD-based) estimates of local genetic correlation, the TWAS approach effectively distinguishes between direct genetic effects on gene expression and indirect LD confounding[21]. In this research, TWAS was conducted utilizing Fusion-based software. Among the most widely used TWAS methods (e.g., PrediXcan, TWAS-Fusion, and SMR) that interrogate causal relationships between gene expression and complex traits[22]. TWAS-Fusion is favored for its unique advantages in our research: 1) GWAS summary data, without relying on individual-level genotypes; 2) Bayesian sparse linear-mixed models excel at capturing cis-regulatory effects. Briefly, Bayesian sparse linear-mixed models[23] were utilized to calculate SNP expression weights for each gene within its 1-Mb cis-regulatory region. The association between predicted gene expression levels and IBS was then estimated using the formula: Z = w·Z/(w·[Lw])[21], where w represents the SNP weight for gene expression prediction, Z denotes the Z-score from IBS GWAS summary data, and L stands for the SNP correlation matrix (i.e., LD structure). Each genetic feature was defined as a contiguous 100,000 bp genomic segment. For the joint model construction, features with a minimum P-value ≤ 0.05 were included; features with an r > 0.9 were considered redundant (identical) and merged, while those with an r < 0.008 were treated as independent to avoid overcounting.

2.3 Gene Expression Omnibus (GEO) datasets

Although FUSION enables the mapping of disease-associated loci identified by TWAS to the transcriptional level, a notable limitation exists in its analytical framework: the transcriptomic data utilized for constructing gene expression weights in FUSION are exclusively derived from normal tissues. Such normal tissue-based expression profiles can only partially recapitulate the transcriptional characteristics of disease states, leading to potential inconsistencies between the predicted gene expression patterns and the actual transcriptional landscape in pathological conditions. To address this constraint and improve the reliability of our findings, we employed previously published transcriptomic data retrieved from the GEO database to further filter the candidate genes identified by FUSION, thereby enhancing the credibility and biological relevance of our study outcomes[24].

**Question2:** The statement regarding “33 significant unduplicated genes” alongside the listing of hundreds of genes per tissue is confusing. It is unclear what “unduplicated” means in this context (e.g., non-redundant across tissues, or differentially expressed?). This needs clearer explanation.

**Response:** We sincerely appreciate the reviewers for pointing out the ambiguity in our descriptions. We have revised, standardized, and refined the relevant content as follows:

We performed FDR correction on the TWAS-identified genes, yielding 33 significant genes after correction; following the removal of duplicates, 27 genes were retained (Figure 2A).

**Question3:** If all genes are significant after FDR correction, the purpose of Figure 2.A is not obvious. The figure legends also require clarification—should the corrected genes across all five tissues amount to five overlapping genes, or ~1,120 combined? This discrepancy should be addressed.

**Response:** We sincerely appreciate the reviewers' comments. We have revised Figure 2: specifically, we removed the content of Figure 2A and retained only Figure 2B. Additionally, we performed functional analyses separately on genes with a TWAS Z-score > 0 and those with a TWAS Z-score < 0, and the updated relevant content is as follows:

3.2 Functional exploration of the TWAS-identified genes associated with IBS

We performed FDR correction on the TWAS-identified genes, yielding 33 significant genes after correction; following the removal of duplicates, 27 genes were retained (Figure 2A). We subjected genes with a TWAS Z value > 0 and Z value < 0 to KEGG and GO enrichment analyses separately (Figure 2B). Results showed that genes with a TWAS Z value > 0 were significantly enriched in pathways including regulation of sodium ion transport, cerebral cortex GABAergic interneuron differentiation, response to histamine, regulation of dopamine receptor signaling pathway, dopamine uptake involved in synaptic transmission, peristalsis, branching morphogenesis of a nerve, negative regulation of ion transport, regulation of cation channel activity, and postsynaptic modulation of chemical synaptic transmission. The activation of sodium ion transport pathways suggests that the upregulation of these genes may be associated with abnormal water-electrolyte metabolism in diarrhea-predominant IBS patients, while the activation of neurotransmitter-related pathways indicates that the upregulation of relevant genes may be linked to abnormalities in neurotransmitter uptake, secretion, and signal transmission in IBS. In contrast, genes with a TWAS Z value < 0 were significantly enriched in pathways such as antigen processing and presentation of endogenous peptide antigen via MHC class I, antigen processing and presentation of endogenous peptide antigen, antigen processing and presentation of endogenous antigen, regulation of integrin activation, positive regulation of macrophage migration, MHC protein complex, MHC protein binding, Natural killer cell mediated cytotoxicity, Allograft rejection, and Autoimmune thyroid disease. The activation of antigen processing and presentation pathways as well as autoimmune-related pathways suggests that the downregulation of these genes may be associated with chronic low-grade inflammation and autoimmune tendency in IBS, while the activation of integrin activation pathways indicates that the downregulation of relevant genes may be related to impaired integrity of the intestinal barrier in IBS.

Regarding the issue of five overlapping genes, the 1,120 genes we used were selected with a threshold of P < 0.05. As shown in Figure 2A, no single gene could be detected across all five tissues after FDR correction. Nevertheless, we still aimed to identify genes with tissue universality. Clinically, the detection of intestinal tissues is obviously limited; therefore, we intended to find blood biomarkers that can reflect the gene expression profile of intestinal tissues through overlapping analysis, which may have greater application value. We have also supplemented the relevant descriptions as follows:

To identify genes co-expressed in different tissues, we performed an overlap analysis of the 1120 significant TWAS-identified genes (P TWAS < 0.05). The Venn diagram suggested that there were five significant TWAS-identified genes associated with IBSamong the five tissues (Figure 2C, Table 1), including MutS homolog 2 (MSH2), DQ alpha 1 (HLA-DQA1), butyrophilin subfamily 3 member A2 (BTN3A2), transmembrane protein 80 (TMEM80), and cathepsin W (CTSW). Detection of intestinal tissues is notably limited in clinical practice; thus, these five overlapping genes are promising candidates as blood biomarkers that can reflect the gene expression profile of intestinal tissues.

**Question4:** While enrichment analysis was performed on the common genes, a comparison of GO/KEGG enrichment for significant genes in each tissue individually would provide additional insights.

Section 3.3 should specify the selection criteria for “enriched terms” and the types of “interactions” used in STRING. Figure 3, especially 3.A, is crowded and difficult to interpret; presenting results for common differentially expressed genes may improve clarity.

**Response:** We sincerely appreciate the reviewers' valuable comments, which have been of great assistance to our study. We have incorporated your two suggestions and significantly revised Figure 3: specifically, we performed Protein-Protein Interaction (PPI) and functional analyses on the differentially expressed genes (DEGs) from three tissues, namely the small intestine, colon, and whole blood. The updated results are as follows:

3.3 Tissue-specific functional analysis

We further performed enrichment analysis separately on the differentially expressed genes (DEGs) in the small intestine, colon, and blood tissues (Figure 3). To further capture the relationships between the terms, a subset of enriched terms was selected and rendered as a network plot, where terms with a similarity > 0.3 are connected by edges. The network was visualized using Cytoscape 5, where each node represents an enriched term and is colored first by its cluster ID and then by its p-value. The differentially expressed genes in the three tissues (colon, small intestine, and blood) exhibit distinct tissue-specific pathways, each focusing on their respective physiological roles and exerting unique biological significance in IBS. For the colon, its unique core pathways include VEGFR2 mediated cell proliferation, MHC class II protein complex assembly, Butanoate metabolism, eicosanoid metabolic process, and progesterone metabolic process, with functional focus on intestinal local immune regulation (e.g., MHC class II-mediated antigen presentation in mucosal immunity), utilization of gut microbiota metabolites (e.g., Butanoate, a key energy source for colonic epithelial cells), and intestinal mucosal cell proliferation; biologically, abnormal Butanoate metabolism in the colon may impair intestinal barrier integrity by reducing energy supply to epithelial cells and weakening tight junctions, thereby exacerbating IBS symptoms like abdominal pain and diarrhea, while dysregulated MHC class II protein complex assembly can disrupt antigen presentation to T cells, leading to mucosal immune imbalance and chronic low-grade inflammation (a core pathological feature of IBS), and abnormal VEGFR2-mediated cell proliferation may hinder colonic mucosal repair, further compromising the intestinal barrier. In the small intestine, the unique pathways are mitochondrial tRNA aminoacylation, sulfur compound biosynthetic process, Ovarian steroidogenesis, Primary ovarian insufficiency, and regulation of leukocyte mediated immunity, focusing on energy metabolism (mitochondrial function, as mitochondrial tRNA aminoacylation is critical for mitochondrial protein synthesis), steroid hormone biosynthesis, and local intestinal immune activation (leukocyte-mediated immune responses); in terms of IBS relevance, mitochondrial tRNA aminoacylation abnormalities can reduce energy production in small intestinal epithelial cells, disrupting nutrient absorption and intestinal motility to cause IBS-related malabsorption and irregular bowel habits, dysregulated leukocyte-mediated immunity can trigger local inflammatory responses to accelerate mucosal damage and enhance visceral hypersensitivity (a key driver of IBS abdominal pain), and abnormal Ovarian steroid metabolism may explain the higher prevalence of IBS in women, as sex hormone fluctuations (e.g., during the menstrual cycle) can exacerbate small intestinal dysfunction. As for the blood, its unique core pathways include Cellular responses to stress, Adaptive Immune System, Membrane Trafficking, endocytosis, Metabolism of RNA, and Ebola virus infection in host, with functional emphasis on systemic immune response (regulation by the adaptive immune system), stress adaptation (cellular stress signaling), and subcellular material transport (membrane trafficking and endocytosis); biologically, abnormal cellular responses to stress in the blood highlight the role of the "brain-gut axis" in IBS, as psychological or environmental stressors can trigger systemic stress signaling via the circulatory system to disrupt intestinal motility and visceral sensitivity, explaining stress-induced exacerbation of IBS symptoms (e.g., increased abdominal pain, altered defecation frequency), dysregulated adaptive immunity indicates a systemic inflammatory state in IBS, where circulating immune cells (e.g., T cells, B cells) and inflammatory factors may infiltrate the intestinal mucosa to amplify local inflammation and worsen barrier dysfunction, and impaired membrane trafficking/endocytosis can disrupt the secretion and uptake of neurotransmitters and inflammatory cytokines in the circulatory system, hindering signal communication between blood and intestinal tissues and perpetuating the "intestinal-systemic" pathological loop in IBS.

**Question5:** In Section 3.4, TWAS and DEGs are reported to share 18 genes. It would be useful to contextualize this relative to the 1,120 TWAS genes, and to indicate their tissue distribution. Similarly, whether the five common TWAS genes are represented in the DEG results and how they are expressed should be clarified.

The reported numbers of unique genes (1101 TWAS vs. 315 DEG) should be corrected and explained. The large differences may reflect tissue- or phenotype-specific factors, which merit discussion.

**Response:** We sincerely appreciate the reviewers' comments and insightful perspectives. In response to the requests from other reviewers, we replaced the original dataset with the higher-quality GEO dataset GSE166869. We have also conducted in-depth reflections on tissue specificity and updated the relevant content as follows:

2.3 Gene Expression Omnibus (GEO) datasets

The GSE166869 dataset was retrieved from the GEO database (https://www.ncbi.nlm.nih.gov/geo/). This study enrolled 26 patients with IBS, including 12 with constipation-predominant IBS (IBS-C) and 14 with diarrhea-predominant IBS (IBS-D), as well as 15 healthy volunteers. Probe-based confocal laser endoscopy was used, and mucosal biopsies of the duodenum and jejunum were obtained for bulk RNA sequencing (RNA-seq). The aim of this study was to identify the small intestinal mechanisms underlying lipid-induced symptoms and rectal hypersensitivity in IBS based on RNA-seq data[25].

3.4 Common genes shared by TWAS and mRNA expression profiling

Differential analysis was performed on the small intestinal tissues of GSE166869, resulting in 24 upregulated DEGs and 17 downregulated DEGs. The intersection of DEGs from the small intestinal tissues of GSE166869 and TWAS-identified significant genes in small intestinal tissues was taken; unfortunately, no overlapping genes were obtained (Figure 4A). Differential analysis was conducted on the colon tissues of GSE166869, yielding 616 upregulated DEGs and 625 downregulated DEGs. Taking the intersection of DEGs from the colon tissues of GSE166869 and TWAS-identified significant genes in colon tissues, 24 overlapping genes were acquired (Figure 4B). The details of these 24 overlapping genes are detailed in Table 2, including 13 upregulated DEGs and 11 downregulated DEGs. KEGG and GO enrichment analyses were performed on the upregulated and downregulated DEGs among the overlapping genes, respectively (Figure 4C). The activation of inflammatory mediators and inflammatory pathways suggests that the upregulation of these genes may be associated with excessive inflammatory activation in irritable bowel syndrome (IBS). The activation of Glutathione metabolism and branched-chain amino acid degradation pathways indicates that the upregulation of relevant genes may be related to oxidative stress in the intestinal cells of IBS patients. The downregulation of MHC class II antigen processing and presentation pathways suggests that the downregulation of relevant genes may be associated with immune recognition defects in IBS. The downregulation of pathways related to negative regulation of cell growth indicates that the downregulation of corresponding genes may be linked to abnormal cell proliferation and impaired mucosal repair capacity in IBS.

Notably, the tissue-specific distribution of TWAS genes and GEO-derived DEGs shows strong consistency: colon-related genes (629 TWAS candidate genes, 1229 DEGs) are substantially more abundant than small intestinal-related genes (241 TWAS candidate genes, 41 DEGs) in both datasets. This discrepancy highlights that the pathological changes of IBS are predominantly concentrated in the colon rather than the small intestine, which is consistent with the clinical manifestation of IBS—abnormal intestinal function mainly involving the colonic segment. Further analysis reveals three key implications: first, the quantitative dominance of colon-related genes reflects the higher genetic and transcriptional regulatory activity in colonic tissues during IBS pathogenesis, suggesting that the colon is the core tissue mediating IBS-related genetic effects and transcriptional dysregulation. Second, the lack of overlapping genes between small intestinal TWAS candidates and GEO-derived small intestinal DEGs may be attributed to the relatively milder pathological alterations in the small intestine of IBS patients, leading to weak transcriptional signals that are difficult to capture. Third, the consistent tissue-specific enrichment pattern between TWAS and GEO datasets enhances the reliability of our findings—both genetic association (TWAS) and transcriptional change (DEGs) analyses independently confirm the colon as the primary site of IBS pathology, providing a solid basis for subsequent targeted exploration of colon-specific biomarkers and therapeutic targets. Regarding the numerical difference between tissue-specific gene sets, the large gap between colon and small intestinal genes (both in TWAS and GEO datasets) further reflects the tissue heterogeneity of IBS genetic regulatory networks. Genetic effects and transcriptional responses during disease progression are not uniformly distributed across intestinal segments; instead, they are concentrated in the colon, which may be related to the colon’s unique physiological functions (e.g., water absorption, fecal formation, and microbial colonization) that make it more susceptible to environmental and genetic perturbations. This tissue-specific feature also explains the low overlap ratio between small intestinal-related gene sets, while the colon-related gene sets show a relatively meaningful overlap (24/629, 3.82% of colon TWAS genes; 24/1229, 1.95% of colon DEGs)—a ratio that is biologically reasonable for complex diseases, considering the multi-factorial nature of IBS pathogenesis.

**Question6:** Figures 4 and 5 require improvement. Figure 4.B shows a healthy sample clustering close to IBS samples—could this be due to normalization or batch effects? Was this also observed in the clustering analysis (Figure 4.D)? Figure 5 would be more informative if it included network analysis outputs such as hub genes or subgraphs.

**Response:** We sincerely appreciate the questions raised by the reviewers. In response to the requests from other reviewers, we replaced the original dataset with the higher-quality GEO dataset GSE166869.

We have updated Figure 5 in light of the reviewers' comments, which includes performing Protein-Protein Interaction (PPI) analysis on the TWAS genes. The updated content is as follows:

3.5 CGSEA of the TWAS-identified genes

We conducted a CGSEA analysis to explore the environmental factors affecting IBS, identifying 1,789 chemicals (Supplementary Information 2), including 112 significant chemicals (FDR< 0.05, |normalized enrichment score, NES|>1). Table 3 lists the top50 compounds ranked by NES. We classified 112 significant chemicals and mapped them to the genes identified by TWAS (Figure 5A). The classifications include Drugs, Nutrients, Organic Compounds, Pollutants/Heavy Metals/Carcinogens, Inorganic Compounds, and Pesticides. The number and proportion of compounds corresponding to each category are shown in Figure 5B. We further performed PPI (Protein-Protein Interaction) analysis on the TWAS-identified genes and used the MCODE plugin to identify 5 key MCODEs in the PPI network (Figure 5C). Additionally, we analyzed the hub genes in the network using the CytoHubba plugin (Figure 5D). Notably, HLA-DQA1 and HLA-DQA2 are not only hub genes but were also identified in our previous analysis.

**Question7:** Table 3 could be enhanced by categorizing chemicals (e.g., drugs, nutrients), which would add interpretability.

**Response:** We sincerely appreciate the reviewers' comments. We have added the compound classification information in Table 3 and Figure 5.

**Question8:** In the discussion, while drug families such as antipsychotics and anti-inflammatories are highlighted, the lack of enrichment for other drugs in the same families is not addressed. A mechanistic discussion comparing enriched vs. non-enriched drugs would strengthen the interpretation.

**Response:** We sincerely appreciate the reviewers' comments. We have further discussed the above issues as follows.

5	Discussion

Health-related quality of life has been significantly reduced in patients with IBS, increasing the disease burden [35]. Unlike many other gastrointestinal disorders, IBS is a symptom-based diagnosis that presents significant challenges in terms of diagnosis, underlying pathophysiology, and management [36]. In addition to gastrointestinal-specific mechanisms, interactions between the digestive system and the nervous system are increasingly recognized among the possible causes of IBS. Therefore, the study of IBS-related gene expression differences can help identify new biomarkers to assist in diagnosis. In addition, studying environmental compounds associated with IBS provides new ideas for identifying risk factors,and opens the possibility of developing preventive strategies based on chemical exposure identification.

We performed TWAS analysis to predict the significant genes expressed in the small intestine, transverse colon, sigmoid colon, whole blood, and peripheral blood. Our results suggest that there are five significant TWAS-identified genes associated with IBS among the five tissues, including MSH2, HLA-DQA1, BTN3A2, TMEM80, and CTSW. A large-scale epidemiological review [37] has demonstrated a significantly higher incidence of colorectal cancer following an IBS diagnosis, with an overall risk ratio of approximately 1.5 and a nearly 6.8-fold increase within the first year after diagnosis, indicating that genetic variations in MSH2 (a gene involved in DNA mismatch repair) and related loci may underlie this predisposition. The low level of inflammation underlying the pathogenesis of IBS has received increasing research attention [38]. HLA-DQA1 (a gene involved in DNA mismatch repair), BTN3A2 (butyrophilin subfamily 3 member A2, involved in immune regulation), TMEM80 (transmembrane protein 80), and CTSW (cathepsin W, a cysteine protease expressed in lymphocytes) are all associated with immune response. We further performed functional enrichment analysis of these TWAS-identified genes. KEGG and GO enrichment analyses showed that the MHC class II protein complex pathway is the main pathway that plays an important role in IBS. Intestinal epithelial cells express MHC class II molecules during homeostasis and inflammation and can produce different functional outcomes [39]; this is consistent with our findings. We also enriched for sex hormone-related pathways, such as steroid binding, which helps to explain the sex bias in IBS [40]. Neurotransmitter-gut interactions are one of the key regulatory mechanisms underlying the pathogenesis of IBS [41], and our study also identified cellular amide metabolic process pathways, which may involve neurotransmitters such as serotonin (5-HT) and gamma-aminobutyric acid (GABA), known to be critical in gut-brain communication. In this study, we determined some new pathways associated with IBS, such as galactosyltransferase, acetylglucosaminyltransferase, and acetylglucosaminyltransferase activities, showing that the role of gut microbes and their metabolites in IBS is not negligible [42].

IBS is a functional disease [43], thus, avoiding exposure to risk factors and alleviating its symptoms may be more important. We extended the classic GSEA approach to detect the association between environmental chemicals and IBS using CGSEA analysis. We identified various chemicals, including drugs, pesticides, organic compounds, inorganic compounds, nutrients, pollutants, heavy metals, and carcinogens. IBS is often comorbid with psychiatric disorders such as anxiety and depression [44]. The gut-brain axis is a bidirectional neurohumoral integrated communication between the microbiota and autonomic nervous system [45]. Gut-brain interaction disorders are associated with upper and lower gastrointestinal food intake-induced symptoms, which may be an emerging mechanism for studying IBS [46]. Imipramine is the most significant compound for NES obtained in our analysis using CGSEA and is also a commonly used clinical antidepressant [47]. Other antipsychotics, such as methylphenidate, flecainide, riluzole, pentylenetetrazole, and chlorpromazine, were also observed in our analysis. It is noteworthy that while antipsychotics as a class were enriched, not all drugs within this family showed significant associations, suggesting specificity in the mechanisms linking certain psychoactive compounds to IBS pathophysiology.For instance, the differential enrichment of specific tricyclic antidepressants (e.g., imipramine) but not others may reflect their distinct pharmacological profiles, such as varying affinities for neurotransmitter transporters or ion channels that modulate gut-brain signaling. Similarly, among anti-inflammatory agents, only a subset was significantly enriched, pointing to distinct inflammatory pathways being relevant. We have enriched the anti-inflammatory class of drugs, including ibuprofen, valdecoxib, geldanamycin, and metribolone, which support the role played by inflammation in IBS from the perspective of gene-compound interactions. The absence of enrichment for other common anti-inflammatory drugs (e.g., aspirin or certain corticosteroids) may indicate that the association is specific to compounds affecting particular molecular targets (e.g., COX-2 inhibition or HSP90 modulation) rather than a broad class effect. Similarly, among anti-inflammatory agents, only a subset was significantly enriched, pointing to distinct inflammatory pathways being relevant. Another group of compounds of interest is flavonoids such as catechin, grape seed proanthocyanidins, and sesame oil. Research [48] has shown that plant polyphenols are beneficial for IBS by reducing oxidative stress, altering gut permeability, and affecting gut enzyme activity and microbes. A recent study reported gastrointestinal distress in humans after exposure to pesticides, which usually manifests as IBS. Our results also showed that pesticides might be related to IBS, indicating that the effect of pesticides on IBS may be a new direction for future research. However, it is important to note that the associations identified between environmental chemicals and IBS in this study are correlational and do not imply causality; further experimental and epidemiological validation is required to establish causal links.

**Question9:** The conclusion is relatively weak and would benefit from more detail, emphasizing the significance of the findings and potential directions for future work.

**Response:** We sincerely appreciate the reviewers' comments. We have re examined this conclusion and accepted your feedback, providing further expanded explanations as follows:

6	Conclusions

This study identified 33 significant genes and 112 environmental chemicals associated with IBS through integrated transcriptomic and chemical-gene interaction analyses. Five key genes (MSH2, HLA-DQA1, BTN3A2, TMEM80, and CTSW) were consistently expressed across all examined tissues, primarily involved in immune-inflammatory pathways and gut-brain interactions. The chemical associations highlight potential environmental risk factors, particularly neuroactive and anti-inflammatory compounds. These findings provide new insights into IBS pathogenesis and identify promising targets for future research on biomarkers and therapeutic strategies.

**Question10:** Supplementary Information 1–2 was not available for review and should be provided.

**Response:** We sincerely appreciate the reviewers for pointing out the issues. We will upload the supplementary files during the revision.

**Question11:** Minor Concerns:

-Several typographical and formatting errors should be corrected. Examples include:

-Section title “2.4. Gene functionalanalyses” → “2.4. Gene functional analyses”

-Section 3.2 “IBSamong” → “3.2. IBS among”

-Section 3.4 “GSE14841DEGs” → “GSE14841 DEGs”

-Figure 4 legend “C: vocanol” → “C: volcano”

-A thorough proofreading will improve readability.

**Response:** We sincerely appreciate the reviewers for pointing out the grammatical and formatting errors in our manuscript—we have corrected all of them. Meanwhile, we will conduct a more thorough proofreading of the manuscript to ensure accuracy.

Reviewer #3: This work from Xinliang Zhao et al. presents an interesting and powerful data analysis implemented with the aim of identifying diagnostic markers and improving the mechanistic understanding of IBS, a syndrome whose etiology is complex and poorly understood.

GENERAL COMMENTS AND SCIENTIFIC APPROACH

The work carried out here should be better highlighted in the writing. The manuscript suffers from a general lack of explanation and detail, whether in terms of the methodology used, the description of the results obtained, or the discussion of the scope of the results.

Five tissues were studied here, including three in the intestine and two in the blood. The TWAS study highlights five significant genes common to all five tissues and bases its discussion and conclusions on these five genes. As the authors' approach aims to provide useful discoveries for both the etiological and mechanistic understanding of IBS and for diagnosis, it would be relevant for them to also search separately for markers specific to the intestine or specific to blood. In particular, one can imagine the diagnostic or even prognostic value of blood biomarkers that can thus be accessed non-invasively. In this regard, the authors should clarify for the reader the definition and difference between peripheral blood and whole blood.

METHODS AND DATA

*Explain and justify the choice of datasets used, particularly the GEO expression dataset comprising only 9 samples.

*The 2 Supplementary Information files cited in the manuscript must have a title and be described in the body of the manuscript.

*Why was an analysis performed to “find DEGs between EGR3 overexpression and negative control groups”? What is the link with this study?

RESULTS

*A general lack of explanation in the captions for figures and tables hinders their comprehension:

• Fig. 1: specify that the red dots on the figure correspond to the significant TWAS genes

• Fig. 2 A-B: 33 genes are mentioned in the text but only 27 are shown in the figure

• Fig 5: The title alone is not sufficient; add explanatory details in the caption.

• Table 1: Specify the nature and origin of the values presented.

*Some passages are difficult to understand:

Results 3.1:

The sentence “A total of 33 significant unduplicated genes (PFDR<0.05; Figure 1), including 241, 392, 390, 337, and 200 genes, were identified in these five tissues, respectively (Supplementary Information 1)” is confusing and needs to be clarified. According to Fig. 1, we understand that TWAS identified a total of 33 significant genes (from the 27+21+13+19+27 red dots) in the five tissues. In this case, what do the “241, 392, 390, 337, and 200 genes” correspond to?

Results 3.2:

Why say “33 genes in adipose tissue” about these genes when adipose tissue is not mentioned anywhere else in the manuscript?

For a better understanding, specify that the 5 significant genes identified by TWAS are common to all 5 tissues.

*The presentation of the results needs to be reworked:

The biological and chemical contributions of the study that appear in the results could be presented in a more detailed and attractive way in order to provide a basis for discussion.

Results 3.2: The paragraph on functional results simply copies and lists the enriched functional terms shown in Fig. 2D, whereas a summary presentation would avoid repeating redundant terms between several of the databases used (e.g., mismatch repair, MHC class II). In addition, the term “positive regulation of isotype switching towards IgG subclasses” is cited in the text, although it does not appear in Fig. 2D.

Results 3.3: As mentioned above, here the list of functional clusters is partially copied as is from the (truncated!) sentences in the figure, instead of presenting the main clusters in a summary form.

Results 3.5: The presentation of the chemicals identified by the study is virtually non-existent (4 lines) and is limited to the number of products without any mention of the different categories of products found.

INTRODUCTION AND DISCUSSION

*Introduction:

The introduction lacks important concepts about IBS that are developed in the discussion:

-Interactions between the digestive system and the nervous system among the possible causes of IBS.

-The possibility of developing an approach to prevent IBS based on the identification of chemicals linked to this syndrome.

*The discussion needs to be reworked:

Revise the following sentences (awkward phrasing): “Health-related quality of life has been significantly reduced in patients with IBS, increasing the disease burden [31]. Unlike many other gastrointestinal disorders, IBS is a symptom-based diagnosis that presents significant challenges in terms of diagnosis, underlying pathophysiology, and management [32].”

Expand the text by better showing the links between the results obtained and the existing literature, in order to highlight the new insights provided by the results. In addition, ensure that the main elements of the results included in the Discussion have been previously mentioned in the Results section.

For clarity, the few genes mentioned in the discussion should be cited by their full names and functions, not just their gene symbols. For example, citing “MSH2 and related loci” as a possible gene explaining the increased risk of colorectal cancer in IBS patients without mentioning that this gene is related to the mismatch repair functions found three times in Figure 2D does not help the reader understand the value of this study.

Similarly, it would be interesting for the authors to explain which neurotransmitters could be involved in IBS, rather than limiting themselves to the single sentence “Neurotransmitter-gut interactions are one of the key regulatory mechanisms underlying the pathogenesis of IBS [37], and our study also identified cellular amide metabolic process pathways.” Which neurotransmitters may actually be involved in this metabolic pathway?

The following sentence refers to an interesting result that is not explicitly mentioned in the Results: “We also enriched for sex hormone-related pathways, such as steroid binding, which helps to explain the sex bias in IBS [36].” The same applies to the classes of chemicals identified, which are not described in the results.

It should be remembered that correlation does not imply causality and that the associations found here between chemicals and IBS should be confirmed by other studies

*Conclusion too short:

Either expand and clarify it or delete it and merge it with the discussion.

FORM AND EDITING

*Quality of figures must be improved:

The figures should be enlarged to fill the entire available width to make them more prominent and easier to read.

Figs. 1, 3, and 4 are difficult to read even when zoomed in; enlarge the characters and improve their definition.

Fig. 1: The color code to the right of each plot is not explained in the figure caption and does not seem useful for understanding the figure. Therefore, remove it or explain it if it is justified. The figure could be made more compact by superimposing the plots on two columns (the three intestinal tissues on the left, the two blood types on the right).

Fig. 3: The list of cluster IDs is incomplete because it is truncated on the right.

Table 3: Specify in the legend the source of the reference indicated in the “chemicals” column.

*Review the use of acronyms:

-Be sure to define acronyms before using them:

Paragraph 2.6: “CTD” is used before its full form, which appears later; the same applies to CGSEA: link the acronym to its full form in the first sentence.

-Define them only once:

There is no need to repeat the full form of IBS; the acronym is sufficient in 2.6 and 3.2. The same applies to “normalized enrichment score,” which is defined in Methods; NES is sufficient in 3.5.

*Avoid repetition in the text: there is no need to repeat the data accession number seven times in the caption for Fig. 4, or to repeat the names of the five tissues twice in the caption for Fig. 1.

*English corrections and wording:

Caption Fig. 1: “transverse/sigmoid colon” and not “transverse/sigmoid colon”

Caption Fig. 2: “shown” instead of “showing”

Caption Fig. 2D: add “the” in “Bar plot of KEGG and GO enrichment of the 5 overlapping genes”

Results 3.2: Correct the sentence “This analysis utilized the Kyoto Encyclopedia of Genes and Genomes (KEGG) and Gene Ontology (GO) frameworks, revealing that the top three pathways exhibit significant enrichment, as illustrated in Figure 2D.” “ to ”This analysis utilized the Kyoto Encyclopedia of Genes and Genomes (KEGG) and Gene Ontology (GO) frameworks, and the top three pathways exhibiting significant enrichment from each database are illustrated in Figure 2D."

Results 3.3: Reword: rather than “We used 1,120 TWAS-significant genes for the PPI analysis and successfully transformed 904 protein-coding genes,” the sentence should be something like “From the 1,120 TWAS-significant genes found, the PPI analysis was successfully applied to 904 protein-coding genes.”

*Review the spacing between words throughout the manuscript: there are words that are too close together and spaces that are unnecessary.

*Inconsistent use of upper and lower case letters, particularly in the lists of functional pathways (Fig. 3) and chemicals (Table 3).

**Reviewer 3:** This work from Xinliang Zhao et al. presents an interesting and powerful data analysis implemented with the aim of identifying diagnostic markers and improving the mechanistic understanding of IBS, a syndrome whose etiology is complex and poorly understood.

GENERAL COMMENTS AND SCIENTIFIC APPROACH

The work carried out here should be better highlighted in the writing. The manuscript suffers from a general lack of explanation and detail, whether in terms of the methodology used, the description of the results obtained, or the discussion of the scope of the results.

Five tissues were studied here, including three in the intestine and two in the blood. The TWAS study highlights five significant genes common to all five tissues and bases its discussion and conclusions on these five genes. As the authors' approach aims to provide useful discoveries for both the etiological and mechanistic understanding of IBS and for diagnosis, it would be relevant for them to also search separately for markers specific to the intestine or specific to blood.

**Response:** Thank you for these precious comments concerning my manuscript. These comments are all valuable and very helpful for revising and improving my paper, as well as the important guiding significance to my researches. We have studied comments carefully and have made corrections which we hope meet with approval. The responds to the reviewer’s comments are as follows:

**Question 1:** In particular, one can imagine the diagnostic or even prognostic value of blood biomarkers that can thus be accessed non-invasively. In this regard, the authors should clarify for the reader the definition and difference between peripheral blood and whole blood.

**Response:** We sincerely appreciate the reviewers for pointing out the inadequacies in our descriptions. We have supplemented the differences between whole blood and peripheral blood as follows:
We specifically analyzed gene expression in the small intestine, transverse colon, sigmoid colon, whole blood (comprising blood cells, plasma, and all other components in the blood), and peripheral blood (blood predominantly composed of blood cells), employing pre-calculated functional weights in conjunction with the IBS GWAS summary data to evaluate gene expression through the predictive model available in FUSION. The gene expression weight panel for the aforementioned tissues was downloaded from the FUSION website (http://gusevlab.org/projects/fusion/).

**Question 2:** Explain and justify the choice of datasets used, particularly the GEO expression dataset comprising only 9 samples.

**Response:** We highly appreciate the reviewers for pointing out the key issue that the data quality of 9 samples may be poor. We replaced the original dataset with the higher-quality GEO dataset GSE166869 and updated the relevant content as follows:

2.3 Gene Expression Omnibus (GEO) datasets

The GSE166869 dataset was retrieved from the GEO database (https://www.ncbi.nlm.nih.gov/geo/). This study enrolled 26 patients with IBS, including 12 with constipation-predominant IBS (IBS-C) and 14 with diarrhea-predominant IBS (IBS-D), as well as 15 healthy volunteers. Probe-based confocal laser endoscopy was used, and mucosal biopsies of the duodenum and jejunum were obtained for bulk RNA sequencing (RNA-seq). The aim of this study was to identify the small intestinal mechanisms underlying lipid-induced symptoms and rectal hypersensitivity in IBS based on RNA-seq data[25].

**Question 3:** Supplementary Information 1–2 was not available for review and should be provided.

**Response:** The 2 Supplementary Information files cited in the manuscript must have a title and be described in the body of the manuscript.

**Question 4:** Why was an analysis performed to “find DEGs between EGR3 overexpression and negative control groups”? What is the link with this study?

**Response:** We sincerely appreciate the reviewers for pointing out the inaccuracies in our description. We have revised the description in the Methods section as follows:

2.4 Gene functionalanalyses

GEO2R [23] was used to find DEGs between diarrhea-irritable bowel syndrome (d-IBS) patients and control groups. P value < 0.05 and |Log2FC| >1 were considered as differentially expressed genes (DEGs). Quality control was performed using principal component analysis (PCA) via the prcomp function and ggplot2 to verify sample grouping. The volcano plot and heatmap analysis were done using R packages[24][25].Functional enrichment analysis of DEGs was conducted with the clusterProfiler package for Gene Ontology (GO) and Kyoto Encyclopedia of Genes and Genomes (KEGG) pathways, with significant terms filtered by adjusted P-value < 0.05(Benjamini-Hochberg method for false discovery rate correction)[26].

**Question 5:** A general lack of explanation in the captions for figures and tables hinders their comprehension:

• Fig. 1: specify that the red dots on the figure correspond to the significant TWAS genes

• Fig. 2 A-B: 33 genes are mentioned in the text but only 27 are shown in the figure

• Fig 5: The title alone is not sufficient; add explanatory details in the caption.

• Table 1: Specify the nature and origin of the values presented.

**Response:** We sincerely appreciate the reviewers for pointing out the general lack of explanations in our figure legends and table captions. We have updated the figure legends and table captions as follows:
Figure 1. Manhattan plots of the association results from the whole transcriptome-wide association study. The horizontal line indicates *P_TWAS_*, with green dots indicating *P_TWAS_*<0.05 and red dots indicating *P_TWAS_*<0.01. Each point represents the genetic predicted expression of a specific gene by the prediction models for small intestine, colon transverse, colon sigmoid, whole blood, and peripheral blood tissues. The x-axis represents the genomic position of the corresponding gene, and the y-axis represents the negative logarithm of the association P-value.

Regarding the issues related to Figure 2, we have provided a comprehensive explanation in the main text as follows:

3.2 Functional exploration of the TWAS-identified genes associated with IBS

We performed FDR correction on the TWAS-identified genes, yielding 33 significant genes after correction; following the removal of duplicates, 27 genes were retained (Figure 2A).

For Figure 5, we have made significant updates, incorporating further analysis of the types of compounds and protein-protein interaction (PPI) analysis of TWAS-related genes. Specific details are as follows:

3.5 CGSEA of the TWAS-identified genes

We conducted a CGSEA analysis to explore the environmental factors affecting IBS, identifying 1,789 chemicals (Supplementary Information 2), including 112 significant chemicals (FDR< 0.05, |normalized enrichment score, NES|>1). Table 3 lists the top50 compounds ranked by NES. We classified 112 significant chemicals and mapped them to the genes identified by TWAS (Figure 5A). The classifications include Drugs, Nutrients, Organic Compounds, Pollutants/Heavy Metals/Carcinogens, Inorganic Compounds, and Pesticides. The number and proportion of compounds corresponding to each category are shown in Figure 5B. We further performed PPI (Protein-Protein Interaction) analysis on the TWAS-identified genes and used the MCODE plugin to identify 5 key MCODEs in the PPI network (Figure 5C). Additionally, we analyzed the hub genes in the network using the CytoHubba plugin (Figure 5D). Notably, HLA-DQA1 and HLA-DQA2 are not only hub genes but were also identified in our previous analysis.

For Table 1, we have supplemented the table caption as follows:

*Nature of values: *P _TWAS_* represents the statistical significance P-value for the association between predicted gene expression levels and irritable bowel syndrome (IBS) in transcriptome-wide association study (TWAS). It reflects the strength of the statistical association between gene expression levels and disease phenotypes, with a smaller value indicating a stronger association.
*Tissue definitions: Small intestine, colon sigmoid, and colon transverse represent intestinal mucosal tissues, while whole blood and peripheral blood represent hematopoietic tissues. These tissues cover the core pathological tissues (intestine) relevant to IBS and potential systemic regulatory tissues (blood).

**Question 6:** Results 3.1: The sentence “A total of 33 significant unduplicated genes (PFDR<0.05; Figure 1), including 241, 392, 390, 337, and 200 genes, were identified in these five tissues, respectively (Supplementary Information 1)” is confusing and needs to be clarified. According to Fig. 1, we understand that TWAS identified a total of 33 significant genes (from the 27+21+13+19+27 red dots) in the five tissues. In this case, what do the “241, 392, 390, 337, and 200 genes” correspond to?

Results 3.2: Why say “33 genes in adipose tissue” about these genes when adipose tissue is not mentioned anywhere else in the manuscript?

For a better understanding, specify that the 5 significant genes identified by TWAS are common to all 5 tissues.

**Response:** We sincerely appreciate the reviewers for pointing out the unclear descriptions in our manuscript. We have made significant revisions to the content of the results sections 3.1 and 3.2. The detailed updated descriptions are as follows:

3.1 TWAS of IBS

TWAS identified 11,000 unduplicated genes from the GWAS summary data: 2,844, 5,261, 4,829, 4,679, and 2,445 genes for the small intestine, transverse colon, sigmoid colon, whole blood, and peripheral blood, respectively (Figure1, Supplementary Information 1).

3.2 Functional exploration of the TWAS-identified genes associated with IBS

We performed FDR correction on the TWAS-identified genes, yielding 33 significant genes after correction; following the removal of duplicates, 27 genes were retained (Figure 2A). We subjected genes with a TWAS Z value > 0 and Z value < 0 to KEGG and GO enrichment analyses separately (Figure 2B). Results showed that genes with a TWAS Z value > 0 were significantly enriched in pathways including regulation of sodium ion transport, cerebral cortex GABAergic interneuron differentiation, response to histamine, regulation of dopamine receptor signaling pathway, dopamine uptake involved in synaptic transmission, peristalsis, branching morphogenesis of a nerve, negative regulation of ion transport, regulation of cation channel activity, and postsynaptic modulation of chemical synaptic transmission. The activation of sodium ion transport pathways suggests that the upregulation of these genes may be associated with abnormal water-electrolyte metabolism in diarrhea-predominant IBS patients, while the activation of neurotransmitter-related pathways indicates that the upregulation of relevant genes may be linked to abnormalities in neurotransmitter uptake, secretion, and signal transmission in IBS. In contrast, genes with a TWAS Z value < 0 were significantly enriched in pathways such as antigen processing and presentation of endogenous peptide antigen via MHC class I, antigen processing and presentation of endogenous peptide antigen, antigen processing and presentation of endogenous antigen, regulation of integrin activation, positive regulation of macrophage migration, MHC protein complex, MHC protein binding, Natural killer cell mediated cytotoxicity, Allograft rejection, and Autoimmune thyroid disease. The activation of antigen processing and presentation pathways as well as autoimmune-related pathways suggests that the downregulation of these genes may be associated with chronic low-grade inflammation and autoimmune tendency in IBS, while the activation of integrin activation pathways indicates that the downregulation of relevant genes may be related to impaired integrity of the intestinal barrier in IBS.

**Question 7:** *The presentation of the results needs to be reworked:

The biological and chemical contributions of the study that appear in the results could be presented in a more detailed and attractive way in order to provide a basis for discussion.

Results 3.2: The paragraph on functional results simply copies and lists the enriched functional terms shown in Fig. 2D, whereas a summary presentation would avoid repeating redundant terms between several of the databases used (e.g., mismatch repair, MHC class II). In addition, the term “positive regulation of isotype switching towards IgG subclasses” is cited in the text, although it does not appear in Fig. 2D.

Results 3.3: As mentioned above, here the list of functional clusters is partially copied as is from the (truncated!) sentences in the figure, instead of presenting the main clusters in a summary form.

Results 3.5: The presentation of the chemicals identified by the study is virtually non-existent (4 lines) and is limited to the number of products without any mention of the different categories of products found.

**Response:** We sincerely appreciate the reviewers for pointing out the unclear descriptions in our manuscript. We have made significant revisions to the content of the results sections 3.2, 3.3 and 3.5. The detailed updated descriptions are as follows:
3.2 Functional exploration of the TWAS-identified genes associated with IBS

We performed FDR correction on the TWAS-identified genes, yielding 33 significant genes after correction; following the removal of duplicates, 27 genes were retained (Figure 2A). We subjected genes with a TWAS Z value > 0 and Z value < 0 to KEGG and GO enrichment analyses separately (Figure 2B). Results showed that genes with a TWAS Z value > 0 were significantly enriched in pathways including regulation of sodium ion transport, cerebral cortex GABAergic interneuron differentiation, response to histamine, regulation of dopamine receptor signaling pathway, dopamine uptake involved in synaptic transmission, peristalsis, branching morphogenesis of a nerve, negative regulation of ion transport, regulation of cation channel activity, and postsynaptic modulation of chemical synaptic transmission. The activation of sodium ion transport pathways suggests that the upregulation of these genes may be associated with abnormal water-electrolyte metabolism in diarrhea-predominant IBS patients, while the activation of neurotransmitter-related pathways indicates that the upregulation of relevant genes may be linked to abnormalities in neurotransmitter uptake, secretion, and signal transmission in IBS. In contrast, genes with a TWAS Z value < 0 were significantly enriched in pathways such as antigen processing and presentation of endogenous peptide antigen via MHC class I, antigen processing and presentation of endogenous peptide antigen, antigen processing and presentation of endogenous antigen, regulation of integrin activation, positive regulation of macrophage migration, MHC protein complex, MHC protein binding, Natural killer cell mediated cytotoxicity, Allograft rejection, and Autoimmune thyroid disease. The activation of antigen processing and presentation pathways as well as autoimmune-related pathways suggests that the downregulation of these genes may be associated with chronic low-grade inflammation and autoimmune tendency in IBS, while the activation of integrin activation pathways indicates that the downregulation of relevant genes may be related to impaired integrity of the intestinal barrier in IBS.

To identify genes co-expressed in different tissues, we performed an overlap analysis of the 1120 significant TWAS-identified genes (P TWAS < 0.05). The Venn diagram suggested that there were five significant TWAS-identified genes associated with IBS among the five tissues (Figure 2C, Table 1), including MutS homolog 2 (MSH2), DQ alpha 1 (HLA-DQA1), butyrophilin subfamily 3 member A2 (BTN3A2), transmembrane protein 80 (TMEM80), and cathepsin W (CTSW). Detection of intestinal tissues is notably limited in clinical practice; thus, these five overlapping genes are promising candidates as blood biomarkers that can reflect the gene expression profile of intestinal tissues. Subsequently, we conducted a functional analysis of the genes associated with IBS identified through TWAS. This analysis utilized the KEGG and GO frameworks, revealing that the top three pathways exhibit significant enrichment, as illustrated in Figure 2D. The KEGG pathway analysis highlighted three notably enriched terms linked to asthma, allograft rejection, and mismatch repair mechanisms. In terms of biological processes, three GO terms were significantly enriched, specifically regarding the antigen receptor-mediated signaling pathway, the positive regulation of isotype switching towards IgG subclasses, and the positive regulation of isotype switching to IgG isotypes. Furthermore, three enriched GO terms related to cellular components were identified, including the MHC class II protein complex, the mismatch repair complex, and the lumen of platelet-dense granules. The molecular functions of the enriched GO terms primarily encompassed binding to mismatch repair complexes, MHC class II receptor activity, and the binding of oxidized DNA. These signal pathways suggest that the pathological processes of IBS are mainly characterized by immune-inflammatory imbalance and autoimmune responses.
3.3 Tissue-specific functional analysis

We further performed enrichment analysis separately on the differentially expressed genes (DEGs) in the small intestine, colon, and blood tissues (Figure 3). To further capture the relationships between the terms, a subset of enriched terms was selected and rendered as a network plot, where terms with a similarity > 0.3 are connected by edges. The network was visualized using Cytoscape 5, where each node represents an enriched term and is colored first by its cluster ID and then by its p-value. The differentially expressed genes in the three tissues (colon, small intestine, and blood) exhibit distinct tissue-specific pathways, each focusing on their respective physiological roles and exerting unique biological significance in IBS. For the colon, its unique core pathways include VEGFR2 mediated cell proliferation, MHC class II protein complex assembly, Butanoate metabolism, eicosanoid metabolic process, and progesterone metabolic process, with functional focus on intestinal local immune regulation (e.g., MHC class II-mediated antigen presentation in mucosal immunity), utilization of gut microbiota metabolites (e.g., Butanoate, a key energy source for colonic epithelial cells), and intestinal mucosal cell proliferation; biologically, abnormal Butanoate metabolism in the colon may impair intestinal barrier integrity by reducing energy supply to epithelial cells and weakening tight junctions, thereby exacerbating IBS symptoms like abdominal pain and diarrhea, while dysregulated MHC class II protein complex assembly can disrupt antigen presentation to T cells, leading to mucosal immune imbalance and chronic low-grade inflammation (a core pathological feature of IBS), and abnormal VEGFR2-mediated cell proliferation may hinder colonic mucosal repair, further compromising the intestinal barrier. In the small intestine, the unique pathways are mitochondrial tRNA aminoacylation, sulfur compound biosynthetic process, Ovarian steroidogenesis, Primary ovarian insufficiency, and regulation of leukocyte mediated immunity, focusing on energy metabolism (mitochondrial function, as mitochondrial tRNA aminoacylation is critical for mitochondrial protein synthesis), steroid hormone biosynthesis, and local intestinal immune activation (leukocyte-mediated immune responses); in terms of IBS relevance, mitochondrial tRNA aminoacylation abnormalities can reduce energy production in small intestinal epithelial cells, disrupting nutrient absorption and intestinal motility to cause IBS-related malabsorption and irregular bowel habits, dysregulated leukocyte-mediated immunity can trigger local inflammatory responses to accelerate mucosal damage and enhance visceral hypersensitivity (a key driver of IBS abdominal pain), and abnormal Ovarian steroid metabolism may explain the higher prevalence of IBS in women, as sex hormone fluctuations (e.g., during the menstrual cycle) can exacerbate small intestinal dysfunction. As for the blood, its unique core pathways include Cellular responses to stress, Adaptive Immune System, Membrane Trafficking, endocytosis, Metabolism of RNA, and Ebola virus infection in host, with functional emphasis on systemic immune response (regulation by the adaptive immune system), stress adaptation (cellular stress signaling), and subcellular material transport (membrane trafficking and endocytosis); biologically, abnormal cellular responses to stress in the blood highlight the role of the "brain-gut axis" in IBS, as psychological or environmental stressors can trigger systemic stress signaling via the circulatory system to disrupt intestinal motility and visceral sensitivity, explaining stress-induced exacerbation of IBS symptoms (e.g., increased abdominal pain, altered defecation frequency), dysregulated adaptive immunity indicates a systemic inflammatory state in IBS, where circulating immune cells (e.g., T cells, B cells) and inflammatory factors may infiltrate the intestinal mucosa to amplify local inflammation and worsen barrier dysfunction, and impaired membrane trafficking/endocytosis can disrupt the secretion and uptake of neurotransmitters and inflammatory cytokines in the circulatory system, hindering signal communication between blood and intestinal tissues and perpetuating the "intestinal-systemic" pathological loop in IBS.

3.5 CGSEA of the TWAS-identified genes

We conducted a CGSEA analysis to explore the environmental factors affecting IBS, identifying 1,789 chemicals (Supplementary Information 2), including 112 significant chemicals (FDR< 0.05, |normalized enrichment score, NES|>1). Table 3 lists the top50 compounds ranked by NES. We classified 112 significant chemicals and mapped them to the genes identified by TWAS (Figure 5A). The classifications include Drugs, Nutrients, Organic Compounds, Pollutants/Heavy Metals/Carcinogens, Inorganic Compounds, and Pesticides. The number and proportion of compounds corresponding to each category are shown in Figure 5B. We further performed PPI (Protein-Protein Interaction) analysis on the TWAS-identified genes and used the MCODE plugin to identify 5 key MCODEs in the PPI network (Figure 5C). Additionally, we analyzed the hub genes in the network using the CytoHubba plugin (Figure 5D). Notably, HLA-DQA1 and HLA-DQA2 are not only hub genes but were also identified in our previous analysis.

**Question 8:** *Introduction:

The introduction lacks important concepts about IBS that are developed in the discussion:

-Interactions between the digestive system and the nervous system among the possible causes of IBS.

-The possibility of developing an approach to prevent IBS based on the identification of chemicals linked to this syndrome.

**Response:** We sincerely appreciate the reviewers' comments. We have added the concept of IBS and the role between the digestive and nervous systems, as follows:

2	Introduction

Irritable bowel syndrome (IBS) represents a significant functional gastrointestinal disorder, exhibiting a prevalence rate ranging from approximately 7% to 21% [1]. Although IBS usually appears in early adulthood [2], its prevalence has significantly increased among children over the last two decades [3]. There are notable gender differences in IBS prevalence, with women in some Western countries being nearly twice as likely to be diagnosed compared to men; this disparity may be even greater in Asian countries [4]. IBS is mainly characterized by recurring abdominal pain and changes in bowel habits [5]. Various factors may contribute to the development of IBS, including genetic factors, changes in gut bacteria, immune system issues, problems with bile salt metabolism, and irregular serotonin levels [6]. Notably, interactions between the digestive system and the nervous system (the gut-brain axis) are increasingly recognized as a key pathophysiological mechanism, potentially explaining the high comorbidity with psychiatric conditions like anxiety and depression Despite these insights, the precise etiology and pathogenesis of IBS remain largely elusive. The syndrome considerably impairs nutritional absorption, growth, and overall quality of life for those affected [7]. Consequently, it is crucial to elucidate the etiology and associated risk factors of IBS to inform and optimize treatment and prevention strategies.

The management of IBS incorporates both pharmacological and non-pharmacological approaches, with the primary aim of alleviating distressing symptoms [8]. Available pharmacotherapeutics for IBS encompass antispasmodics, laxatives, antidiarrheals, analgesics, and probiotics. Nevertheless, the effectiveness of many of these medications is limited, with several studies indicating that their benefits do not significantly surpass those of a placebo [9].As a result, there has been a growing emphasis on non-pharmacological interventions for IBS in recent years [10]. Current clinical guidelines advocate for the use of soluble fiber to address overall IBS symptoms, while antispasmodic agents are presently available in the United States for symptom management [11]. Although dietary factors can exacerbate IBS, they also play a critical role in symptom relief [12]. Moreover, various environmental compounds are linked to intestinal health in patients with IBS [13]. Research has been conducted to explore the interconnections among complex diseases, genetic factors, and environmental chemicals by evaluating the interactions between genetic predispositions and environmental influences [14, 15]. Therefore, understanding the relationship between environmental chemicals and IBS is vital for both treatment and prevention of the syndrome. Identifying specific chemicals associated with IBS could pave the way for novel preventive approaches based on exposure reduction, opening the possibility of developing preventive strategies based on chemical exposure identification.

**Question 9:** *The discussion needs to be reworked:

Revise the following sentences (awkward phrasing): “Health-related quality of life has been significantly reduced in patients with IBS, increasing the disease burden [31]. Unlike many other gastrointestinal disorders, IBS is a symptom-based diagnosis that presents significant challenges in terms of diagnosis, underlying pathophysiology, and management [32].”

Expand the text by better showing the links between the results obtained and the existing literature, in order to highlight the new insights provided by the results. In addition, ensure that the main elements of the results included in the Discussion have been previously mentioned in the Results section.

For clarity, the few genes mentioned in the discussion should be cited by their full names and functions, not just their gene symbols. For example, citing “MSH2 and related loci” as a possible gene explaining the increased risk of colorectal cancer in IBS patients without mentioning that this gene is related to the mismatch repair functions found three times in Figure 2D does not help the reader understand the value of this study.

Similarly, it would be interesting for the authors to explain which neurotransmitters could be involved in IBS, rather than limiting themselves to the single sentence “Neurotransmitter-gut interactions are one of the key regulatory mechanisms underlying the pathogenesis of IBS [37], and our study also identified cellular amide metabolic process pathways.” Which neurotransmitters may actually be involved in this metabolic pathway?

The following sentence refers to an interesting result that is not explicitly mentioned in the Results: “We also enriched for sex hormone-related pathways, such as steroid binding, which helps to explain the sex bias in IBS [36].” The same applies to the classes of chemicals identified, which are not described in the results.

It should be remembered that correlation does not imply causality and that the associations found here between chemicals and IBS should be confirmed by other studies.

**Response:** We sincerely appreciate the reviewers' comments. Many of the suggestions you have put forward have given us great inspiration, and we have made the following modifications:

5	Discussion

The health-related quality of life of IBS patients is significantly reduced, increasing the disease burden [35]. Unlike many other gastrointestinal diseases, IBS is a symptom based diagnosis that presents significant challenges in terms of diagnosis, potential pathophysiology, and management [36]. In addition to gastrointestinal-specific mechanisms, interactions between the digestive system and the nervous system are increasingly recognized among the possible causes of IBS. Therefore, the study of IBS-related gene expression differences can help identify new biomarkers to assist in diagnosis. In addition, studying environmental compounds associated with IBS provides new ideas for identifying risk factors,and opens the possibility of developing preventive strategies based on chemical exposure identification.

We performed TWAS analysis to predict the significant genes expressed in the small intestine, transverse colon, sigmoid colon, whole blood, and peripheral blood. Our results suggest that there are five significant TWAS-identified genes associated with IBS among the five tissues, including MSH2, HLA-DQA1, BTN3A2, TMEM80, and CTSW. A large-scale epidemiological review [37] has demonstrated a significantly higher incidence of colorectal cancer following an IBS diagnosis, with an overall risk ratio of approximately 1.5 and a nearly 6.8-fold increase within the first year after diagnosis, indicating that genetic variations in MSH2 (a gene involved in DNA mismatch repair) and related loci may underlie this predisposition. The low level of inflammation underlying the pathogenesis of IBS has received increasing research attention [38]. HLA-DQA1 (a gene involved in DNA mismatch repair), BTN3A2 (butyrophilin subfamily 3 member A2, involved in immune regulation), TMEM80 (transmembrane protein 80), and CTSW (cathepsin W, a cysteine protease expressed in lymphocytes) are all associated with immune response. We further performed functional enrichment analysis of these TWAS-identified genes. KEGG and GO enrichment analyses showed that the MHC class II protein complex pathway is the main pathway that plays an important role in IBS. Intestinal epithelial cells express MHC class II molecules during homeostasis and inflammation and can produce different functional outcomes [39]; this is consistent with our findings. We also enriched for sex hormone-related pathways, such as steroid binding, which helps to explain the sex bias in IBS [40]. Neurotransmitter-gut interactions are one of the key regulatory mechanisms underlying the pathogenesis of IBS [41], and our study also identified cellular amide metabolic process pathways, which may involve neurotransmitters such as serotonin (5-HT) and gamma-aminobutyric acid (GABA), known to be critical in gut-brain communication. In this study, we determined some new pathways associated with IBS, such as galactosyltransferase, acetylglucosaminyltransferase, and acetylglucosaminyltransferase activities, showing that the role of gut microbes and their metabolites in IBS is not negligible [42].

**Question 10:** *Conclusion too short:

Either expand and clarify it or delete it and merge it with the discussion.

**Response:** We sincerely appreciate the reviewers' comments. We have expanded and revised the conclusion as follows:

6	Conclusions

This study identified 33 significant genes and 112 environmental chemicals associated with IBS through integrated transcriptomic and chemical-gene interaction analyses. Five key genes (MSH2, HLA-DQA1, BTN3A2, TMEM80, and CTSW) were consistently expressed across all examined tissues, primarily involved in immune-inflammatory pathways and gut-brain interactions. The chemical associations highlight potential environmental risk factors, particularly neuroactive and anti-inflammatory compounds. These findings provide new insights into IBS pathogenesis and identify promising targets for future research on biomarkers and therapeutic strategies.

**Question 11:** *Quality of figures must be improved:

The figures should be enlarged to fill the entire available width to make them more prominent and easier to read.

Figs. 1, 3, and 4 are difficult to read even when zoomed in; enlarge the characters and improve their definition.

Fig. 1: The color code to the right of each plot is not explained in the figure caption and does not seem useful for understanding the figure. Therefore, remove it or explain it if it is justified. The figure could be made more compact by superimposing the plots on two columns (the three intestinal tissues on the left, the two blood types on the right).

Fig. 3: The list of cluster IDs is incomplete because it is truncated on the right.

Table 3: Specify in the legend the source of the reference indicated in the “chemicals” column.

**Response:** We sincerely appreciate the reviewers for pointing out the issue of poor image quality. We have made significant adjustments to all the images. In addition, when submitting the manuscript, we will additionally upload higher-resolution images (e.g., TIFF format) to facilitate the editors' use of images with better quality.

For Table 3, relevant annotations have been supplemented as follows:

*The compound IDs and names were retrieved from the Comparative Toxicogenomics Database (CTD, http://ctdbase.org/downloads/), and the PubChem database (https://pubchem.ncbi.nlm.nih.gov/) was used to further verify the compound names. Compound classifications were jointly identified by three researchers with pharmacological or chemical backgrounds. Classifications approved by more than two researchers were included.

**Question 12:** *Review the use of acronyms:

-Be sure to define acronyms before using them:

Paragraph 2.6: “CTD” is used before its full form, which appears later; the same applies to CGSEA: link the acronym to its full form in the first sentence.

-Define them only once:

There is no need to repeat the full form of IBS; the acronym is sufficient in 2.6 and 3.2. The same applies to “normalized enrichment score,” which is defined in Methods; NES is sufficient in 3.5.

**Response:** We sincerely appreciate the reviewers for their careful and patient review. Thank you for pointing out the oversights in the use of abbreviations in our manuscript. We have standardized the use of abbreviations throughout the manuscript.

**Question 13:** Avoid repetition in the text: there is no need to repeat the data accession number seven times in the caption for Fig. 4, or to repeat the names of the five tissues twice in the caption for Fig. 1.

**Response:** We sincerely appreciate the reviewers for pointing out the issue of poor image quality. We have made significant adjustments to all the images.

**Question 14:** English corrections and wording:

Caption Fig. 1: “transverse/sigmoid colon” and not “transverse/sigmoid colon”

Caption Fig. 2: “shown” instead of “showing”

Caption Fig. 2D: add “the” in “Bar plot of KEGG and GO enrichment of the 5 overlapping genes”

Results 3.2: Correct the sentence “This analysis utilized the Kyoto Encyclopedia of Genes and Genomes (KEGG) and Gene Ontology (GO) frameworks, revealing that the top three pathways exhibit significant enrichment, as illustrated in Figure 2D.” “ to ”This analysis utilized the Kyoto Encyclopedia of Genes and Genomes (KEGG) and Gene Ontology (GO) frameworks, and the top three pathways exhibiting significant enrichment from each database are illustrated in Figure 2D."

Results 3.3: Reword: rather than “We used 1,120 TWAS-significant genes for the PPI analysis and successfully transformed 904 protein-coding genes,” the sentence should be something like “From the 1,120 TWAS-significant genes found, the PPI analysis was successfully applied to 904 protein-coding genes.”

*Review the spacing between words throughout the manuscript: there are words that are too close together and spaces that are unnecessary.

*Inconsistent use of upper and lower case letters, particularly in the lists of functional pathways (Fig. 3) and chemicals (Table 3).

**Response:** We sincerely appreciate the reviewers for their careful and patient review. Thank you for pointing out the oversights in the use of abbreviations in our manuscript. We have strengthened the manuscript review and addressed the grammatical issues in it.
